# Human Osteoarthritic Chondrocytes Express Nineteen Different TRP-Genes—TRPA1 and TRPM8 as Potential Drug Targets

**DOI:** 10.3390/ijms241210057

**Published:** 2023-06-13

**Authors:** Leevi Halonen, Antti Pemmari, Elina Nummenmaa, Mari Hämäläinen, Teemu Moilanen, Katriina Vuolteenaho, Eeva Moilanen

**Affiliations:** 1 The Immunopharmacology Research Group, Faculty of Medicine and Health Technology, Tampere University and Tampere University Hospital, 33014 Tampere, Finland; leevi.halonen@tuni.fi (L.H.); antti.pemmari@tuni.fi (A.P.); elina.nummenmaa@tuni.fi (E.N.); mari.hamalainen@tuni.fi (M.H.); teemu.moilanen@coxa.fi (T.M.); katriina.vuolteenaho@tuni.fi (K.V.); 2 Coxa Hospital for Joint Replacement, 33520 Tampere, Finland

**Keywords:** osteoarthritis, chondrocyte, TRPA1, TRPM8, TPR ion channel, glucocorticoid, non-steroidal anti-inflammatory drug, menthol

## Abstract

Transient receptor potential (TRP) ion channels are expressed in neuronal and some non-neuronal cells and are involved particularly in pain and thermosensation. We previously showed that TRPA1 is functionally expressed in human osteoarthritic (OA) chondrocytes and mediates inflammation, cartilage degradation, and pain in monosodium-iodoacetate-induced experimental OA. In the present study, we explored the expression of TRP-channels in primary human OA chondrocytes and investigated whether drugs used in the treatment of OA, ibuprofen and glucocorticoids, have effects on TRP-channel expression. OA cartilage was obtained from knee replacement surgery and chondrocytes were isolated with enzyme digestion. NGS analysis showed the expression of 19 TRP-genes in OA chondrocytes, with TRPM7, TRPV4, TRPC1, and TRPM8 having the highest counts in unstimulated cells. These results were verified with RT-PCR in samples from a different group of patients. Interleukin-1β (IL-1β) significantly increased TRPA1 expression, while TRPM8 and TRPC1 expression was decreased, and TRPM7 and TRPV4 expression remained unaffected. Furthermore, dexamethasone attenuated the effect of IL-1β on TRPA1 and TRPM8 expression. The TRPM8 and TRPA1 agonist menthol increased the expression of the cartilage-degrading enzymes MMP-1, MMP-3, and MMP-13 and the inflammatory factors iNOS and IL-6 in OA chondrocytes. In conclusion, human OA chondrocytes express 19 different TRP-genes, of which the significant TRPM8 expression is a novel finding. Dexamethasone attenuated IL-1β-induced TRPA1 expression. Interestingly, the TRPM8 and TRPA1 agonist menthol increased MMP expression. These results support the concept of TRPA1 and TRMP8 as potential novel drug targets in arthritis.

## 1. Introduction

Transient receptor potential (TRP) channels are a superfamily of cation channels involved in thermosensation, pain, and many other vital functions [1]. They have been widely studied in neurons but are now known to be expressed and functional also in many non-neuronal cells [1]. The significance of TRP channels was acknowledged with the Nobel Prize in 2021 [1].

Originally discovered in drosophila in 1969 [2], TRP channels were later found in mammals with TRP canonical 1 (TRPC1) as the first human TRP channel discovered [3]. At present, 28 TRP channels have been identified [1]. Since their discovery, TRP channels have been the subject of a vast amount of pharmacological research across multiple different therapeutic areas and indications [4] such as respiratory disease [5], oncology [6], pain management [7], and dermatology [8].

We have previously shown that TRP ankyrin 1 (TRPA1) is expressed in human OA chondrocytes. TRPA1 activation increases the expression of OA-related inflammatory and catabolic factors such as interleukin-6 (IL-6), matrix metalloproteinases (MMPs), and prostaglandin E_2_ [9,10]. TRPA1 also mediates inflammation, joint pain, and cartilage degradation in the monosodium iodoacetate-induced OA-model [11]. However, not so much is known about the other TRP channels in OA.

OA is the most common joint disease globally. It is a slowly progressing inflammatory disease that affects the entire joint. The main symptoms are pain and loss of function. Ultimately, the progression of OA leads to the deterioration of the cartilage in the affected joint [12]. The current pharmacological treatment of OA is symptomatic; there is an urgent need for drugs that could retard, prevent or reverse the cartilage damage in OA [12,13]. Non-steroidal anti-inflammatory drugs (NSAIDs) are the most commonly used drugs to treat OA pain. During the exacerbation of OA symptoms, an intra-articular glucocorticoid injection can be administered to alleviate inflammation and pain [13]. Given the role of TRP channels in inflammation and pain, and our previous findings on TRPA1 in OA, these channels present as potential drug targets.

In addition to TRPA1, mechanosensitive channels TRPV4 and TRPC1 as well as some other TRP channels have been studied in human chondrocytes in a predetermined manner [14]. In the present study, we conducted a genome-wide expression analysis in order to produce more comprehensive data on the expression of TRP channels in human OA chondrocytes. We also investigated whether drugs commonly used in the treatment of OA, ibuprofen or glucocorticoids, have effects on TRP channel expression.

## 2. Results

**Figure 1:**
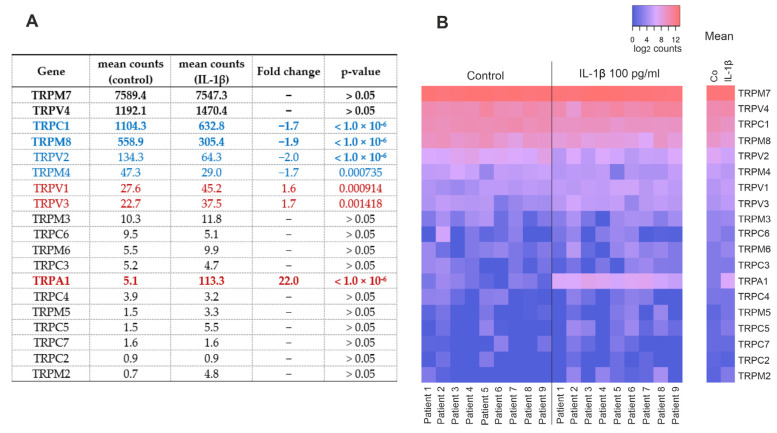
TRP-channels detected in primary human OA chondrocytes by NGS analysis. Chondrocytes were treated with IL-1β (100 pg/mL) for 24 h, after which total RNA was extracted. (**A**) Table showing the expression of TRP channels with or without IL-1β treatment. Blue = downregulated TRP channels, red = upregulated TRP channels, and bold = genes chosen for RT-PCR. (**B**) Heatmap showing the expression of TRP channels in each patient with red signifying higher and blue lower expression. Counts in (**A**) are DeSeq2-normalized mean counts, fold changes were calculated from raw counts and presented in (**A**) if the change was statistically significant with a *p*-value of <0.05. *p*-values are false discovery rate (FDR)-adjusted. Counts in (**B**) are log_2_-transformed DeSeq2-normalized counts with counts ≤ 1 treated as 0. n = 9 patients.

We analyzed the expression of TRP genes in human OA chondrocytes from our recently published NGS analysis [15]. Nineteen TRP channels were expressed at detectable levels, with TRPM7, TRPV4, TRPC1, and TRPM8 having the highest counts (Figure 1). Stimulation with the OA-related cytokine IL-1β increased the expression of TRPA1, TRPV1, and TRPV3, while the expression levels of TRPV2, TRPM8, TRPC1, and TRPM4 were reduced roughly by half compared to the unstimulated chondrocytes (Figure 1).

**Figure 2:**
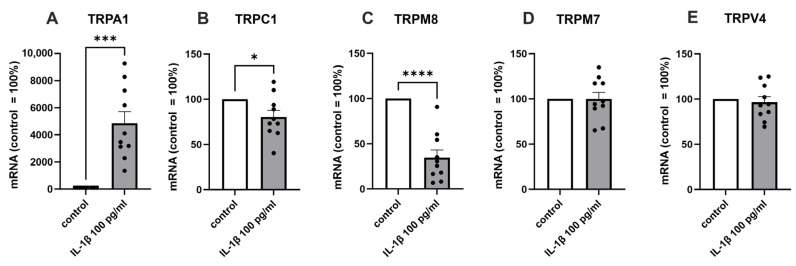
RT-PCR confirmation of the expression of the selected TRP channels in a different group of patients. Chondrocytes were treated with IL-1β (100 pg/mL) for 24 h, after which total RNA was extracted. The expression of TRPs was measured by RT-PCR and normalized against GAPDH. Results are expressed as the mean + SEM (standard error of the mean) and dots represent individual data points. Statistical significance was calculated with paired *t*-test, n = 10 patients. * *p* < 0.05, *** *p* < 0.001, **** *p* < 0.0001.

In order to verify the NGS results, we analyzed the TRP channels with the highest expression levels (TRPM7, TRPV4, TRPC1, TRPM8, Figure 1) in a different group of patients using RT-PCR. In addition, we measured the expression of TRPA1, which we have earlier shown in OA chondrocytes [9] and found to be involved in experimental OA [11]. In accordance with the NGS results, IL-1β significantly increased TRPA1 expression, while the expression of TRPC1 and TRPM8 was decreased, and that of TRPM7 and TRPV4 expression levels remained unaltered (Figure 2).

**Figure 3:**
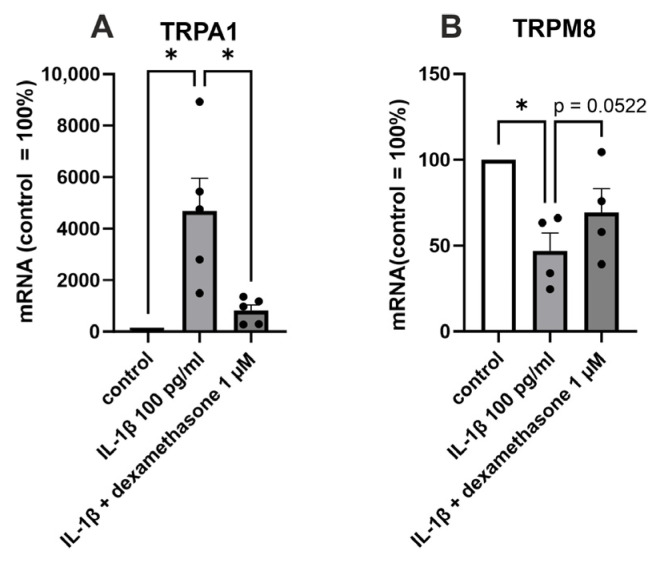
The effect of dexamethasone on TRPA1 and TRPM8 expression in primary human OA chondrocytes. Chondrocytes were treated with IL-1β (100 pg/mL) or a combination of IL-1β (100 pg/mL) and dexamethasone (1 µM) for 24 h, after which total RNA was extracted. The expression of TRPA1 and TRPM8 was measured by RT-PCR and normalized against GAPDH. Results are expressed as the mean + SEM (standard error of the mean) and dots represent individual data points. Statistical significance was calculated with repeated measures ANOVA followed by Dunnett post-test, n = 5 patients (**A**) or n = 4 patients (**B**). * *p* < 0.05.

We then aimed to investigate whether drugs used to treat OA pain and inflammation, ibuprofen and glucocorticoids, affect TRP channel expression in OA chondrocytes. The glucocorticoid dexamethasone partly reversed the IL-1β-induced increase in TRPA1 (*p* < 0.05) and decrease in TRPM8 (*p* = 0.0522) expression (Figure 3). Whereas dexamethasone did not alter the expression of the other measured TRP channels (TRPC1, TRPM7, or TRPV4) in OA chondrocytes. Ibuprofen had no effect on any of the measured TRP channels (TRPA1, TRPC1, TRPM8, TRPM7, or TRPV4).

**Figure 4:**
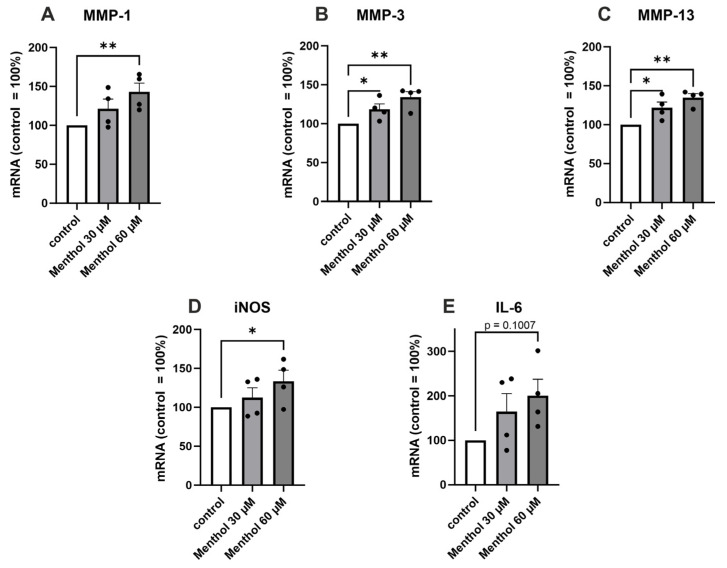
The effect of the TRPM8 and TRPA1 agonist menthol on the expression of MMP-1, MMP-3, MMP-13, iNOS, and IL-6 in primary human OA chondrocytes. Chondrocytes were treated with menthol (30 and 60 µM) for 24 h, after which total RNA was extracted. The expression of MMPs (**A**–**C**), iNOS (**D**), and IL-6 (**E**) was measured by RT-PCR and normalized against GAPDH. Results are expressed as the mean + SEM (standard error of the mean) and dots represent individual data points. Statistical significance was calculated with repeated measures ANOVA with Dunnett post-test, n = 4 patients. * *p* < 0.05, ** *p* < 0.01.

We also preliminarily investigated the effects of the TRPM8 and TRPA1 agonist menthol on the OA-related cartilage-degrading MMPs and the pro-inflammatory factors iNOS and IL-6. Menthol (60 µM) caused a statistically significant increase in the expression of MMP-1, MMP-3, MMP-13, and iNOS. Menthol also showed an increasing trend in the expression of IL-6 (*p* = 0.1007) (Figure 4).

## 3. Discussion

In the present study, we explored TRP channels in a genome-wide expression analysis (GWEA) of human OA chondrocytes [15], which showed the expression of 19 different TRP genes. As novel findings, we report here that TRPM8 is expressed in human OA chondrocytes and that TRPA1 is the most responsive of the TRP channels to stimulation with the OA-related cytokine IL-1β. We also found that the TRPM8 and TRPA1 agonist menthol caused an increase in the expression of the cartilage-degrading enzymes MMP-1, MMP-3, and MMP-13. Dexamethasone partly reversed the effects of IL-1β on TRPA1 and TRPM8 expression. Apart from this, dexamethasone and ibuprofen had no effect on the expression of the other measured TRP channels.

The expression of twelve predetermined TRP channels in primary human OA chondrocytes has previously been studied by Gavenis et al. [14] with RT-PCR. However, GWEA has not previously been used, enabling us to explore the expression of all TRP channels. TRPV6 and TRPM1 were investigated but not detected in the study by Gavenis, which is in accordance with the findings of our study. Expression of TRPC4, TRPC5, and TRPV6 was not detected in the study by Gavenis; however, their expression was found in our NGS data, although with low count values. All other TRP genes shown to be expressed in chondrocytes by Gavenis et al. were also detected in the present analysis. In addition, we found the expression of seven TRP channels that have not been investigated previously.

TRPM8 has been studied in rat models of rheumatoid arthritis [16], but our study is the first to show TRPM8 expression in human OA chondrocytes. We also detected expression of TRPV4 and TRPC1, which have previously been shown to contribute to chondrocyte functions: TRPV4 to regulate chondrogenesis [17] and act as a mechanoreceptor [18], and TRPC1 to respond to mechanical stimuli [19] in chondrocytes. TRPM7, also known as TRP-PLIK and TRPLC7, was the most highly expressed TRP channel in the present study, and this is in accordance with the known ubiquitous expression of this TRP gene with ion channel and kinase activities [20,21].

Glucocorticoids are used as anti-inflammatory drugs in various indications. In exacerbations of OA, intra-articular glucocorticoid injections are recommended to alleviate inflammation and pain in knee OA [13]. In our previous study, dexamethasone was found to downregulate the expression of TRPA1 in primary human chondrocytes [22] and in line with that study, the downregulating effect was also detected here. Interestingly, dexamethasone also tended to inhibit the effect of IL-1β on TRPM8 expression.

After discovering TRPM8 and TRPA1 expression in chondrocytes, we aimed to investigate if menthol, a known agonist of both these channels [23], might have an effect on the expression of OA-related factors. Interestingly, menthol was found to increase the expression of cartilage-degrading MMP enzymes as well as proinflammatory factors iNOS and IL-6. These effects are probably mediated, at least partly, through the activation of the TRPM8 channel, although menthol concentrations used in this study (30 and 60 µM) were lower than the EC50 of 185–196 µM demonstrated in the literature [24,25]. The effect could also be mediated via TRPA1 activation as menthol has been shown to act as a TRPA1 agonist at 10–100 µM concentrations, while higher concentrations are reported to reversibly block TRPA1 activation [26]. These findings are of interest and warrant further studies as menthol is a common constituent in the ointments used in the treatment of OA pain.

The present study showed that human OA chondrocytes express multiple TRP genes, of which TRPM8 and TRPA1 were found to be the most interesting ones. For the first time, we reported here the expression of the menthol receptor TRPM8 in OA chondrocytes. Among the expressed TRP channels, TRPA1 stands out as the most responsive one to inflammatory stimulation, and its induced expression could be downregulated by dexamethasone. Furthermore, menthol, an agonist to both TRPM8 and TRPA1 channels, increased the expression of OA-related factors MMPs, iNOS, and IL-6. These findings, together with the previous data, point to TRPA1 and TRPM8 channels as potential drug targets in osteoarthritis.

## 4. Materials and Methods

Cartilage samples were obtained from 28 OA patients [18 (64%) females, age 67.8 ± 1.6 years, body mass index (BMI) 29.6 ± 1.2 kg/m^2^; mean ± SEM (standard error of the mean)] undergoing knee replacement surgery in Coxa Hospital for Joint Replacement, Tampere, Finland. Chondrocytes were isolated by enzyme digestion as described before [15]. Each experiment was conducted with samples from distinct patients. In the first experiment, primary chondrocytes were cultured alone or with the OA-related cytokine IL-1β (100 pg/mL) before conducting the NGS/RNA-Seq analysis (n = 9 patients). The results were confirmed with RT-PCR in a distinct group of patients (n = 10 patients). In the second experiment, the cells were cultured with IL-1β combined with either ibuprofen (10 µM) or dexamethasone (1 µM; n = 5 patients). For the third experiment, the cells were cultured with menthol (30 and 60 µM; n = 4 patients). Cell culture, NGS/RNA-Seq, and RT-PCR were carried out as described previously [15,27].

## Data Availability

All relevant data to support the findings and conclusion of the study are included in the manuscript.
